# Hypoglycaemia combined with mild hypokalaemia reduces the heart rate and causes abnormal pacemaker activity in a computational model of a human sinoatrial cell

**DOI:** 10.1098/rsif.2021.0612

**Published:** 2021-11-24

**Authors:** Alan Bernjak, Ahmed Iqbal, Simon R. Heller, Richard H. Clayton

**Affiliations:** ^1^ Department of Oncology and Metabolism, University of Sheffield, Medical School, Beech Hill Road, Sheffield S10 2RX, UK; ^2^ INSIGNEO Institute for in silico Medicine, University of Sheffield, Sheffield, UK; ^3^ Sheffield Teaching Hospitals NHS Foundation Trust, Sheffield, UK; ^4^ Department of Computer Science, University of Sheffield, Sheffield, UK

**Keywords:** human sinoatrial node, computational model, hypoglycaemia, heart rate, hypokalaemia, bradycardia

## Abstract

Low blood glucose, hypoglycaemia, has been implicated as a possible contributing factor to sudden cardiac death (SCD) in people with diabetes but it is challenging to investigate in clinical studies. We hypothesized the effects of hypoglycaemia on the sinoatrial node (SAN) in the heart to be a candidate mechanism and adapted a computational model of the human SAN action potential developed by Fabbri *et al.*, to investigate the effects of hypoglycaemia on the pacemaker rate. Using Latin hypercube sampling, we combined the effects of low glucose (LG) on the human ether-a-go-go-related gene channel with reduced blood potassium, hypokalaemia, and added sympathetic and parasympathetic stimulus. We showed that hypoglycaemia on its own causes a small decrease in heart rate but there was also a marked decrease in heart rate when combined with hypokalaemia. The effect of the sympathetic stimulus was diminished, causing a smaller increase in heart rate, with LG and hypokalaemia compared to normoglycaemia. By contrast, the effect of the parasympathetic stimulus was enhanced, causing a greater decrease in heart rate. We therefore demonstrate a potential mechanistic explanation for hypoglycaemia-induced bradycardia and show that sinus arrest is a plausible mechanism for SCD in people with diabetes.

## 1. Introduction

Blood glucose levels are elevated in both type 1 and type 2 diabetes. Insulin is used to therapeutically lower high glucose in individuals with type 1 and long-standing type 2 diabetes; however, this commonly results in iatrogenic hypoglycaemia, one major barrier in achieving and maintaining normal glucose control in diabetes [1]. Insulin causes glucose to fall, but in individuals with diabetes, insulin levels are reduced due to auto-immune damage to the pancreatic *β* cells (type 1 diabetes) or a combination of impaired insulin secretion and action (type 2 diabetes). In both types of diabetes, exogenous insulin is used to lower glucose therapeutically but due to the limitations of current insulin delivery, insulin replacement is unphysiological. It is challenging to achieve stable basal concentrations and those with insulin-treated diabetes are subject to both hyper- and hypoglycaemia. Attempts to mimic normal glucose levels through intensive insulin therapy increase the risk of severe hypoglycaemic episodes. The effects of hypoglycaemia on the cardiovascular system have only recently emerged and severe hypoglycaemia has been associated with increased mortality in randomized trials [2,3]. In one of the trials, intensive glucose therapy resulted in a sixfold increase in severe hypoglycaemia [2]. Over one-third of deaths were attributed to sudden cardiac death (SCD), raising the possible link between hypoglycaemia and cardiac arrhythmias.

Indeed, there is growing evidence in recent clinical and animal studies that hypoglycaemia might contribute to malignant cardiac arrhythmias leading to SCD. Severe hypoglycaemia in response to glucose therapy was associated with an increased risk of arrhythmic death in individuals with type 2 diabetes [3]. Spontaneous nocturnal hypoglycaemia was also implicated in the deaths of young people with type 1 diabetes. The term ‘dead in bed syndrome’ was introduced to describe these unexpected deaths [4], which were attributed to SCD. Subsequent studies reported a relatively large proportion of unexplained deaths in this population [5,6]. Although rare in absolute numbers, SCD is 10-fold higher in young people with type 1 diabetes compared to those without diabetes [7]. In observational studies, we and others reported an increased risk of arrhythmias, including slow heart rate, bradycardia, during spontaneous hypoglycaemia versus normoglycaemia (NG) [8–11]. These arrhythmias could be related to abnormal cardiac repolarization observed in spontaneous [12] and experimental hypoglycaemia studies, where hypoglycaemia was induced by an infusion of insulin [13–15]. Experimental hypoglycaemia in rats can cause lethal cardiac arrhythmias, although at glucose levels significantly lower compared to those in human studies [16].

Possible hypoglycaemia-induced proarrhythmic mechanisms include the direct effect of low extracellular glucose on the myocardial cell, which can block potassium channels that carry an outward current during repolarization [17]. This causes a prolongation in the cardiac action potential (AP) as well as lengthening of the QT interval duration in the ECG through a mechanism identical to that in proarrhythmic medications [18]. In addition, the counterregulatory sympathoadrenal activation in response to hypoglycaemia causes a release of adrenaline [19], which in turn lowers serum potassium concentration [20] and increases intracellular calcium concentration [21]. Combined, these can cause further QT interval prolongation and calcium overload and lead to proarrhythmogenic electrical instability [21]. Abnormal autonomic responses which are common in diabetes could cause further risk in these individuals.

The autonomic response to falling glucose levels includes sympathetic and parasympathetic activation, with the latter limiting the increase in heart rate during hypoglycaemia. However, bradycardia was also observed during spontaneous [22] and experimental hypoglycaemia [23]. In our observational studies, we detected increased rates of bradycardia during hypoglycaemia compared to NG in some individuals with diabetes [8,10,11]. Bradycardia was also common during experimental hypoglycaemia in rats [24]. These observations suggest that bradycardia might be an additional risk factor during hypoglycaemia, possibly by the development of early afterdepolarizations leading to ventricular tachycardia, particularly in the presence of hypokalaemia [25]. Our focus in this study was therefore on the effect of hypoglycaemia on the sinoatrial node (SAN), the heart's natural pacemaker.

Hypoglycaemia studies present challenges to investigators. While hypoglycaemia and cardiac arrhythmias are relatively common, SCD is rare, suggesting the involvement of additional factors, including variable and dysfunctional autonomic responses and impaired awareness of hypoglycaemia. Observational clinical hypoglycaemia studies are limited by an inability to measure electrolytes and catecholamines during spontaneous episodes of hypoglycaemia. Experimental studies on the other hand while more controlled require supra-physiological concentrations of insulin to induce hypoglycaemia, rarely encountered in clinical practice. In addition, a greater fall in potassium in these studies is not representative of what happens in people with diabetes in the ‘free living’ condition. Animal models offer an alternative approach but species differences in the hormonal responses to hypoglycaemia and ensuing cardiac electrophysiological responses mean there are limitations in drawing conclusions from these data.

In this study, we have therefore used a computational model to overcome some of these limitations. There are currently no computational models that describe the electrophysiological responses to hypoglycaemia, and in this study, we adapted a biophysically detailed computational model of a human SAN cell electrophysiology to examine the effect of hypoglycaemia on pacemaker rate.

## 2. Methodology

### 2.1. Model of the human sinoatrial node action potential

We used a computational model of the human SAN AP, developed by Fabbri *et al.* [26]. This model was in turn adapted from a computational rabbit SAN model, developed by Severi *et al.* [27] by incorporating experimental data from human SAN cells. These included updated cell capacitance and dimension data, a new formulation of the *I*_f_ current, new steady-state activation and updated conductances of delayed rectifier K^+^ currents (*I*_Kr_ and *I*_Ks_) and added *I*_Kur_ current formulation. Parameters, for which human experimental data were not available, were fitted using automatic parameter optimization. The modulation of the pacemaker rate in response to autonomic stimulation was similarly adapted from the rabbit model, where acetylcholine- and isoprenaline-induced variations were introduced to mimic the activation of the sympathetic (increased rate) and parasympathetic (decreased rate) nervous system, respectively. Further details about the model are provided in the electronic supplementary material.

We ran simulations in MATLAB (The MathWorks, Inc., Natick, MA, USA) vR2017a, using automatically generated code for the Fabbri model obtained from the CellML platform (www.cellml.org). For solving numerical differentiation, we used the ‘ode15s' function with relative error tolerance 10^−7^, absolute error tolerance 10^−6^ and a maximum step size of 10^−3^ s. We ran the simulations for 60 s and used the last beat to calculate the features of AP waveforms and for figures. We confirmed the stability of the main features of the AP waveform (electronic supplementary material, figure S1).

### 2.2. Features of sinoatrial action potential waveform

**Figure 1.**
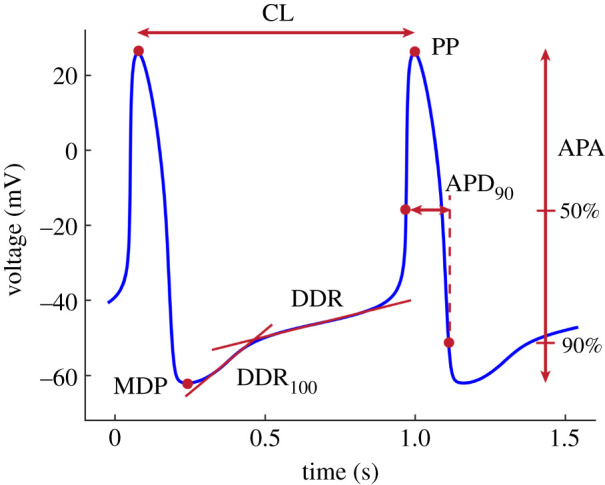
Features of the sinoatrial AP waveform. CL—cycle length; PP—peak potential; APA—AP amplitude; MDP—maximum diastolic potential; DDR—diastolic depolarization rate; DDR_100_—early diastolic depolarization rate; APD_90_—AP duration at 90% repolarization.

We characterized the sinoatrial AP waveform using a set of parameters presented by Fabbri *et al.*, and we calculated them based on the guidelines for standardization of AP parameters [28]. The following parameters were calculated: CL—cycle length of the pacemaker activity, defined as the duration between the peaks of two consecutive APs; PP—peak action potential, peak (maximum value) of the AP; APA—AP amplitude, defined as the difference between the maximum and minimum AP potentials; MDP—maximum diastolic potential preceding the PP, defined as the most negative repolarization potential; DDR—diastolic depolarization rate; DDR_100_—diastolic depolarization rate over the first 100 ms of diastolic depolarization; APD_90_—AP duration at 90% repolarization (figure 1).

The main parameter of our interest was CL. We explored the changes induced by hypoglycaemia and the main features of AP responsible for these changes.

### 2.3. Effect of low extracellular glucose—experimental data

In an experimental study, Zhang *et al.* demonstrated a direct effect of low extracellular glucose concentration (*Glu*o) on the rapid delayed rectifier potassium current (*I*_Kr_) [17]. Using a whole-cell patch clamp, they showed an impairment in the function of the corresponding human ether-a-go-go-related gene (hERG) channel, proportional to the reduction in *Glu*o. Zhang *et al.* used human embryonic kidney cells (HEK293 cells), and in this study, we assume that electrophysiological properties and responses of the hERG channels are similar in the heart, where hERG is highly expressed.

Zhang *et al.* demonstrated two separate mechanisms by which reduced *Glu*o alters the function of the hERG channel: a reduction in *I*_Kr_ current density and a shift in its current–voltage (I–V) relationship. We digitized their graphs to numerically quantify the extent of changes. We further describe the effect size for both mechanisms and how we incorporated them in our model.

#### 2.3.1. Reduced *I*_Kr_ current density

Zhang *et al.* presented experimental data for *I*_Kr_ I–V relationships at various *Gluo*: *Glu*o = 5 (NG), 2.5, 1 and 0 mmol l^−1^. They demonstrated a 10–20% reduction in current density at *Glu*o = 2.5 mmol l^−1^ compared to 5 mmol l^−1^, a 20–30% reduction at 1 mmol l^−1^ and a 50% reduction at 0 mmol l^−1^. The current density decreased proportionally with decreased *Glu*o, but the reduction was not constant across the voltage range. In our model, we incorporated the drop in current density by decreasing the conductance (*g*_Kr_) of *I*_Kr_. For easier interpretation, *g*_Kr_ in our model represents normalized conductance, a multiplier of maximum conductance (4.2 nS in the Fabbri model). We decreased *g*_Kr_ from 1 (maximum conductance, no effect of glucose) to 0 to investigate the overall response of the model. We then varied *g*_Kr_ within the [1, 0.7] range (100 to 70% of maximum conductance) to consider only physiologically relevant glucose concentrations, based on the above data.

#### 2.3.2. Shift in the *I*_Kr_ current–voltage relationship (IVshift)

Zhang *et al.* demonstrated that the *I*_Kr_ I–V relationship shifted towards more negative potentials with reduced *Glu*o. Specifically, they reported a −10 mV shift at *Glu*o = 0 versus 5 mmol l^−1^. There was a similar −10 mV shift in normalized conductance. I–V relationships were not shown for other *Glu*o, but they performed additional experiments under conditions with corrected osmolarity at *Glu*o = 1 mmol l^−1^. In these experiments, they measured a −3 mV shift in normalized conductance at *Glu*o = 1 versus 5 mmol l^−1^ and for the purpose of our study, we assumed that the shift in I–V curves is similar. This is since the shifts in the I–V relationship and conductance were similar in the experiment without correction for osmolarity. To model physiologically relevant effects of hypoglycaemia, we varied IVshift within [−3, 0] mV range but we explored shifts below −3 mV to test the overall response of the model.

We extracted all relevant data by digitizing the graphs, and we measured the shifts at 50% I–V curve or conductance amplitude. In the Fabbri model, *I*_Kr_ is formulated as a function of membrane potential by combining the activation and inactivation kinetics. To model the shift in the I–V curve, we incorporated IVshift into the membrane potential variables of all equations describing the activation and inactivation relationships and their corresponding time constants.

### 2.4. Latin hypercube sampling

We used Latin hypercube sampling for simulations where two or more input parameters were randomly selected from a predefined range. The sampling was performed by using the ‘lhsdesign’ algorithm in MATLAB. The variable input parameters included *g*_Kr_, IVshift and extracellular potassium concentration (*K*o).

### 2.5. Outline of the study

We investigated the individual effect of each parameter *g*_Kr_, IVshift and *K*o on CL and features of the AP waveform. For each parameter, the modelling was performed for physiologically relevant values as well as outside this range to test the behaviour of the model and to identify input values that produce meaningful APs. We also looked at the combined effects of these parameters when their values were within physiologically relevant ranges. Using Latin hypercube sampling, we varied *g*_Kr_ (*g*_Kr_ is a multiplier of maximum conductance) between 0.7 and 1, IVshift between −3 and 0 mV and *K*o between 3.0 and 4.0 mmol l^−1^. *K*o at 4.0 mmol l^−1^ represents normal *K*o while *K*o = 3.0 mmol l^−1^ indicates moderate hypokalaemia. Finally, we checked the combined effect of these parameters with additional activation of either the sympathetic or parasympathetic autonomic system. The current model allows only one of them to be activated at any time.

## 3. Results

### 3.1. Baseline sinoatrial action potential waveform

Fabbri *et al.* fitted and optimized their model at *K*o = 5.4 mmol l^−1^, resulting in CL = 0.81 s (rate = 74 beats per minute (bpm)). For the purpose of our study, we changed the baseline *K*o to 4.0 mmol l^−1^, closer to the normal plasma potassium range in humans which is between 3.5 and 5 mmol l^−1^ [29]. This resulted in prolonged CL = 0.92 s (13.5% increase, 65 versus 74 bpm), which was mostly caused by decreased DDR_100_ (40.1 versus 56.0, −28%) and lower MDP (−62.0 versus −58.9 mV, −5%). The AP waveform at NG and *K*o = 4.0 mmol l^−1^ is presented in figure 1, and its features at *K*o = 5.4 and *K*o = 4.0 mmol l^−1^ are given in table 1. Throughout the results section, percentage changes in parameters are given compared to baseline levels at *K*o = 4.0 mmol l^−1^.

**Table 1.** Features of the AP waveform (rows 4 and onwards) calculated using a combination of input parameters (rows 1 to 3) representing NG, LG and a combination of LG and low potassium. Baseline values of input parameters (italics), their extreme values and their combinations are considered. We changed the baseline *K*o = 5.4 mmol l^−1^ in the Fabbri model to *K*o = 4.0 mmol l^−1^.

	NG			LG			LG + low *K*o		
*g*_Kr_	*1*	*1*	*1*	0.7	*1*	0.7	0.7	*1*	0.7
*IVshift* (*mV*)	*0*	*0*	*0*	*0*	−3	−3	*0*	−3	−3
*Ko* (*mmol l*^−1^)	5.4	*4.0*	3.0	*4.0*	*4.0*	*4.0*	3.0	3.0	3.0
CL (s)	0.81	*0.92*	0.94	0.75	1.20	0.93	0.85	1.33	1.04
PP (mV)	26.4	*25.9*	25.5	27.3	25.7	27.5	27.1	24.8	27.1
APA (mV)	85.3	*87.9*	91.3	84.2	92.5	87.8	86.4	96.1	90.4
MDP (mV)	−58.9	*−62.0*	−65.8	−56.9	−66.8	−60.3	−59.3	−71.3	−63.3
APD_90_ (s)	0.15	*0.14*	0.14	0.17	0.15	0.18	0.17	0.15	0.17
DDR	21.6	*19.0*	20.6	25.1	10.9	18.3	21.9	8.4	16.2
DDR_100_	56.0	*40.1*	35.4	51.0	34.1	52.2	44.9	35.3	33.7

### 3.2. Effect of low glucose

#### 3.2.1. Reduced conductance of the hERG channel

**Figure 2.**
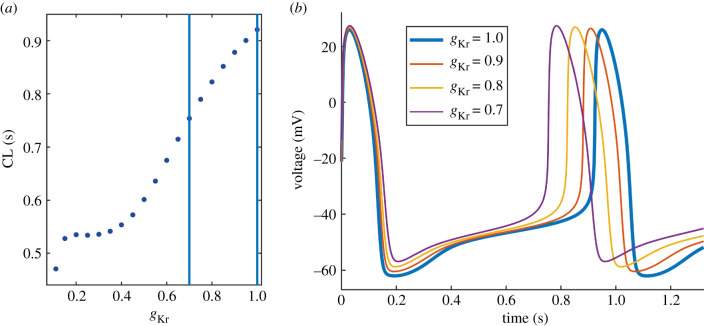
Effect of reduced *g*_Kr_ on the AP waveform. (*a*) Relationship between *g*_Kr_ and CL. Vertical lines indicate the *g*_Kr_ range at clinically relevant glucose concentrations. (*b*) AP waveforms demonstrating shortened CL (increased rate), prolonged APD, elevated MDP and increased DDR with a decrease in *g*_Kr_.

We reduced *g*_Kr_ (*g*_Kr_ is a multiplier of maximum conductance) from 1 (maximum conductance) to 0 in steps of 0.05. The model failed to generate spontaneous AP activity at *g*_Kr_ < 0.11. The relationship between *g*_Kr_ and CL is presented in figure 2*a*. CL shortens with decreased *g*_Kr_ and the relationship is approximately linear down to *g*_Kr_ = 0.4, followed by a plateau. The minimum CL at *g*_Kr_ = 0.11 is 0.47 s (−49%). The vertical blue lines indicate the [0.7, 1] range, which represents our estimated reduction in *g*_Kr_ at clinically relevant reduced glucose concentrations. CL at *g*_Kr_ = 0.7 is 0.75 s (−18%), APD is prolonged (+21%), the MDP potential is elevated (+8%) and the diastolic depolarization rates DDR and DDR_100_ are increased (+32% and +27%) (table 1). Examples of the corresponding AP waveforms are presented in figure 2*b*.

#### 3.2.2. Negative shift in *I*_Kr_ current–voltage relationship

**Figure 3.**
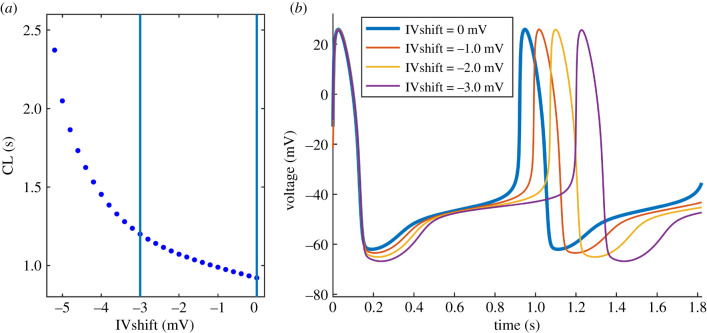
Effect of negative IVshift on the AP waveforms. (*a*) Relationship between CL and IVshift. The blue vertical lines indicate our estimated range of IVshift at clinically relevant glucose concentrations. (*b*) AP waveforms showing gradual CL prolongation with IVshift, mostly caused by decreased MDP and DDR/DDR_100_.

We introduced a negative shift (towards more negative potentials) in the I–V relationship (IVshift), starting from 0 mV in steps of −0.1 mV. CL exponentially increases with negative IVshift (figure 3*a*) and the longest valid CL is obtained at −5.4 mV (CL = 5.94 s). The blue vertical lines indicate the [−3, 0] mV range which represents our estimated shift at clinically relevant glucose concentrations. CL at IVshift = −3 mV equals 1.2 s (+30%) and the prolongation is mostly caused by decreased MDP (−8%) and depolarization rates DDR (−43%) and DDR_100_ (−15%) (table 1). The corresponding AP waveforms are presented in figure 3*b*.

#### 3.2.3. Combined effects of reduced *g*_Kr_ and negative IVshift

**Figure 4.**
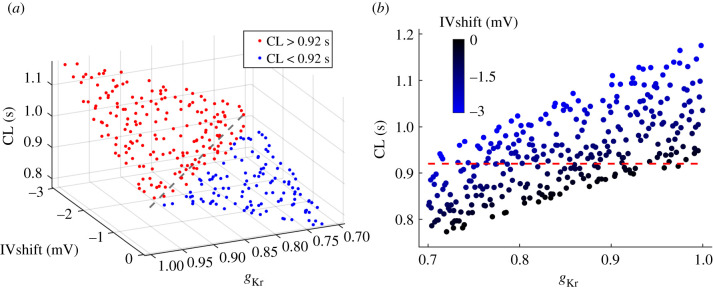
Combined effects of reduced *g*_Kr_ ([0.7, 1]) and negative IVshift ([−3, 0] mV) on CL, using Latin hypercube sampling (*n* = 300). (*a*) CL as a function of *g*_Kr_ and IVshift. Red data indicate CL prolongation and blue data CL shortening versus baseline (CL = 0.92 s). The grey dashed line indicates a linear change in both parameters. (*b*) A projection to the *g*_Kr_ axis reveals gradual shortening of CL with reduced *g*_Kr_. At each *g*_Kr_, CL is prolonged with negative IVshift. The red dashed line shows baseline CL.

We combined the effects of reduced *g*_Kr_ and negative IVshift by simultaneously sampling *g*_Kr_ from [0.7, 1] and IVshift from [−3, 0] mV ranges. We used Latin hypercube sampling to generate 300 pairs of random and independent input variables for 300 evaluations of the model. The resulting CLs are presented in figure 4. Red data indicate CL prolongation and blue data CL shortening versus baseline (CL = 0.92 s) (figure 4*a*). CLs are longest at baseline *g*_Kr_ (*g*_Kr_ = 1) combined with maximum IVshift (IVshift = −3 mV) and are shortest at *g*_Kr_ = 0.7 combined with baseline IVshift (0 mV). The grey dashed line indicates a simultaneous linear change in both parameters. CLs along this line are approximately constant and are slightly longer than baseline CL (approx. 0.93 s, +1%). In figure 4*b*, a projection of CL values to the *g*_Kr_ axis shows a gradual shortening of CL with decreased *g*_Kr_, and for each *g*_Kr_, CL is prolonged with the negative IVshift. The maximum and minimum CLs obtained with these parameters are 1.2 s (+30%) and 0.75 s (−18%), respectively (table 1).

##### 3.2.3.1. Features of the action potential waveform

The individual effects of reduced *g*_Kr_ and negative IVshift produce CL changes in opposite directions (figures 2 and 3) and their combined effects cancel each other out to some degree, producing a slight CL prolongation. A similar opposite effect is obtained with other AP features, including APA, MDP and DDR. For example, baseline MDP equals −62.0 mV (table 1) and the combined effects of *g*_Kr_ = 0.7 and IVshift = −3 mV slightly raise MDP to −60.3 mV (+3%). Individual changes in *g*_Kr_ and IVshift, however, produce a bigger difference compared to baseline with minimum/maximum MDP values −66.8 mV (−8%) and −56.9 mV (+8%), respectively. On the other hand, PP, APD_90_ and DDR_100_ are predominantly affected by one of the input parameters but not by the other. For these AP features, the combined changes in *g*_Kr_ and IVshift produce values outside the range of their individual effects. For example, APD_90_ is prolonged at *g*_Kr_ = 0.7 (APD_90_ = 0.17 s, +21%) but with added IVshift = −3 mV, there is a further prolongation (APD_90_ = 0.18 s, +29%). Maximum values of PP, ADP_90_ and DDR_100_ are achieved when both *g*_Kr_ and IVshift are at the maximum of their ranges.

### 3.3. Effect of low extracellular potassium

**Figure 5.**
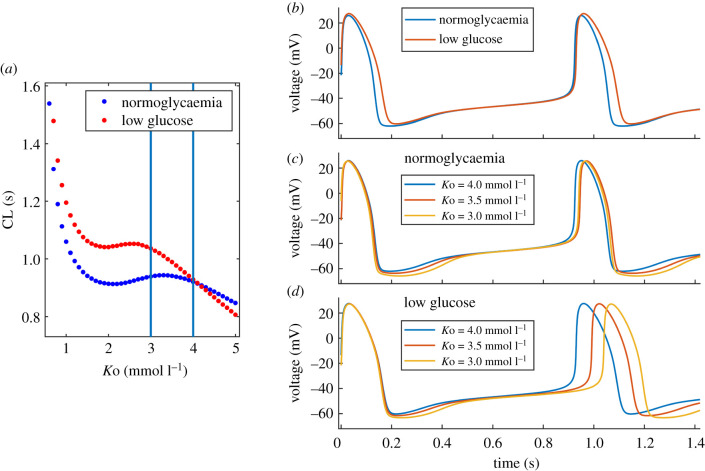
Effect of reduced *K*o on CL. (*a*) Relationship between *K*o and CL at NG (*g*_Kr_ = 1, IVshift = 0 mV, blue) and at LG (*g*_Kr_ = 0.7, IVshift = −3 mV, red). (*b*) AP waveforms at NG and LG (both at *K*o = 4 mmol l^−1^). (*c*) Effect of reduced *K*o on AP waveforms is small during NG. (*d*) Reduced *K*o combined with LG produces a relatively big prolongation in CL.

**Figure 6.**
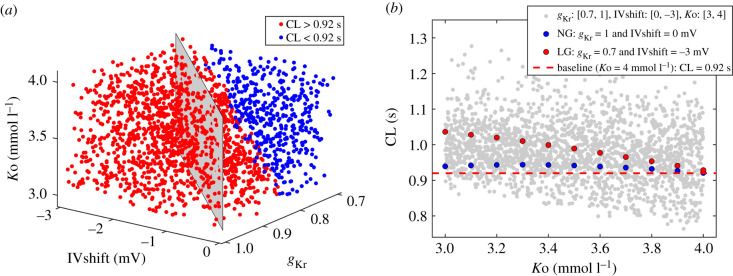
Combined effects of LG and low potassium on CL using randomly sampled *g*_Kr_ ([0.7, 1]), IVshift ([−3, 0] mV) and *K*o ([3, 4] mmol l^−1^). (*a*) Red data indicate CL prolongation and blue data CL shortening versus baseline (CL = 0.92 s). A linear change in *g*_Kr_ and IVshift (grey panel) causes CL prolongation which increases with decreased *K*o. (*b*) Range of CL values for random input parameters (grey). Blue data show the relationship with *K*o at NG and red data at LG when *g*_Kr_ and IVshift are fixed at their extremes (*g*_Kr_ = 0.7, IVshift = −3 mV). Latin hypercube sampling, *n* = 2000.

With the other input parameters at baseline (NG), we lowered *K*o from 5 mmol l^−1^ in steps of −0.1 mmol l^−1^. The relationship between *K*o and CL is presented in figure 6*a* in blue colour. The lowest *K*o that produces periodic pacemaker activity is 0.6 mmol l^−1^ (CL = 1.54 s, +67%). CL is slightly prolonged with the decrease in *K*o from 4 mmol l^−1^ to 3 mmol l^−1^ (blue vertical lines) and reaches a local maximum of 0.94 s (+2%) at 3.3 mmol l^−1^. The prolongation is mainly caused by more negative MDP (−6%) and decreased DDR_100_ (−12%) (figure 5*c* and table 1).

### 3.4. Combined effects of low glucose concentration and potassium concentration

We first combined the effects of low *Glu*o and *K*o by fixing *g*_Kr_ and IVshift at the maximum of their ranges: *g*_Kr_ = 0.7, IVshift = −3 mV and by reducing *K*o in a stepwise manner. Second, we randomly sampled all three parameters (*g*_Kr_, IVshift and *K*o) from their predefined ranges.

#### 3.4.1. Fixed *g*_Kr_ and IVshift, combined with stepwise reduced *K*o

With *g*_Kr_ and IVshift fixed at 0.7 and −3 mV, respectively, we decreased *K*o from 5 mmol l^−1^ in steps of −0.1 mmol l^−1^. The relationship between CL and *K*o is presented in figure 5*a* (red). In contrast with NG (blue), the relationship is steeper for *K*o between 3 and 5 mmol l^−1^, and there is a local maximum at *K*o = 2.6 mmol l^−1^ (CL = 1.05 s, +14%). At *K*o = 4 mmol l^−1^, CL is similar at NG and low glucose (LG) (0.92 s versus 0.93 s, +1%) but with reduced *K*o = 3 mmol l^−1^, there is a notable prolongation in CL at LG (CL = 1.04 s, +13%) versus NG (CL = 0.94 s, +2%). The corresponding AP waveforms are presented in figure 5*b–d*. LG causes APD prolongation (0.18 s, +29%) with a minor effect on CL (0.93 s, +1%) (figure 5*b*). Reduced potassium at NG produces a slight prolongation in CL (figure 5*c*) but the prolongation is substantially greater when combined with LG (figure 5*d*) (CL data are given above).

#### 3.4.2. Randomly sampled *g*_Kr_, IVshift and potassium concentration

We randomly varied *g*_Kr_ = [0.7, 1], IVshift = [−3, 0] mV and *K*o = [3, 4] mmol l^−1^ using Latin hypercube sampling by generating 2000 independent evaluations of the model. The resulting CLs are presented in figure 6. In (figure 6*a*), red data designate the area of input parameters which cause CL prolongation and blue data the area resulting in CL shortening versus baseline. The grey panel indicates a linear change in *g*_Kr_ and IVshift. At *K*o = 4 mmol l^−1^, the model generates both prolongation and shortening of CL. With reduced *K*o, the number of cases resulting in CL shortening is decreasing proportionally and at *K*o = 3 mmol l^−1^, these are confined to the subspace *g*_Kr_ < 0.8 and IVshift > −1 mV. In (figure 6*b*), absolute values of CL are presented (grey) as a function of *K*o. There is a relatively broad spread of CL but also a systematic CL prolongation with reduced *K*o. At *K*o = 3 mmol l^−1^, most of the evaluations of the model result in CL prolongation versus baseline (red dashed line). For reference, data at NG are shown (blue), as well as at LG with *g*_Kr_ and IVshift fixed at 0.7 and −3 mV, respectively (red). The shortest CL is 0.76 s (−17%) at *g*_Kr_ = 0.7, IVshift = 0 mV and *K*o = 3.92 mmol l^−1^, and the longest CL is 1.28 s (+39%) at 1, −3 mV and 3.1 mmol l^−1^, respectively. By combining the effects of randomly sampled input parameters, CL stays within the range obtained from parameters at the extreme ends of their predefined ranges (table 1).

### 3.5. Autonomic modulation

We investigated the effect of autonomic modulation by separately including in the model either sympathetic or parasympathetic activity. In both scenarios, we looked at the combined responses of autonomic activity, LG and low potassium. To model LG, we first fixed *g*_Kr_ and IVshift at the extreme ends of their ranges. Second, we explored all possible outcomes by randomly sampling all input parameters (*g*_Kr_, IVshift and *K*o).

#### 3.5.1. Fixed *g*_Kr_ and IVshift

**Figure 7.**
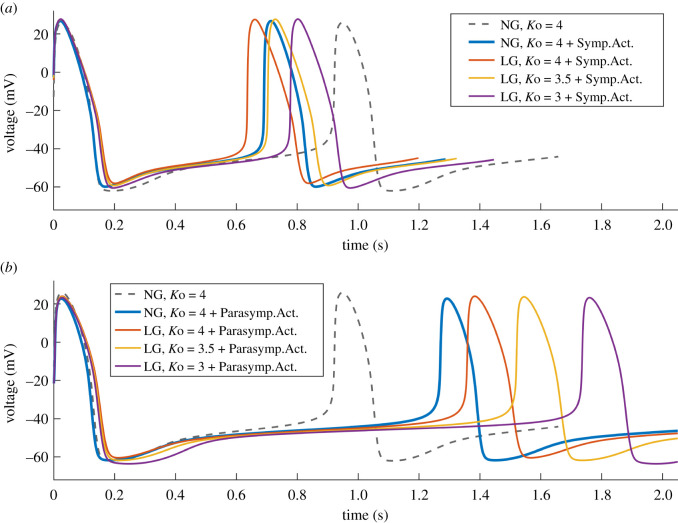
AP waveforms at NG and LG (*g*_Kr_ = 0.7 and IVshift = −3 mV) in combination with reduced *K*o and activation of either (*a*) sympathetic activity (Symp.Act.) or (*b*) parasympathetic activity (Parasymp.Act.). In both cases, reduced *K*o at LG causes gradual CL prolongation.

At NG and *K*o = 4 mmol l^−1^, an addition of sympathetic activity shortens the CL from 0.92 s to 0.69 s (−25%). In figure 7*a*, the grey dashed line shows the AP at NG without and the blue line with added sympathetic activity. LG (*g*_Kr_ = 0.7 and IVshift = −3 mV) combined with sympathetic activity further shortens the CL (0.64 s, −30%, red line). An additional reduction in *K*o causes CL prolongation, reaching 0.70 s (−24%) at *K*o = 3.5 mmol l^−1^ (yellow) and 0.78 s (−15%) at *K*o = 3 mmol l^−1^ (purple).

**Figure 8.**
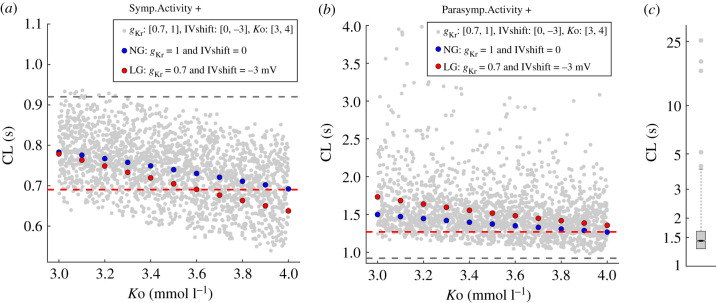
CL with randomly sampled *g*_Kr_ [0.7, 1], IVshift [−3, 0]mV and *K*o [3, 4]mmol l^−1^, using Latin hypercube sampling (*N* = 2000) with the addition of either (*a*) sympathetic activity or (*b*,*c*) parasympathetic activity. Blue and red data present the relationship between CL and *K*o at NG and at LG (*g*_Kr_ = 0.7, IVshift = −3 mV), respectively. Outlier CL values are not shown in (*b*). The distribution of the full range is presented on logarithmic axis in (*c*). Some combinations of input parameters combined with parasympathetic activity did not produce valid pacemaker activity (data not shown). The grey dashed lines show baseline CL at NG (CL = 0.92 s). The red dashed lines show CL at NG combined with (*a*) sympathetic activity (CL = 0.69 s) and (*b*) parasympathetic activity (CL = 1.27 s).

An addition of parasympathetic activity at NG causes CL prolongation from 0.92 s (figure 7*b*, grey dashed line) to 1.27 s (+38%) (blue). LG combined with parasympathetic activity further prolongs the CL (1.36 s, +48%, red). An additional reduction in *K*o causes a further relatively big prolongation: CL = 1.52 s (+65%) at *K*o = 3.5 mmol l^−1^ (yellow) and CL = 1.73 s (+88%) at *K*o = 3 mmol l^−1^ (purple). The features of the AP waveforms are given in table 2, and the relationship between CL and *K*o at NG and LG is presented in figure 8 using blue and red colours, respectively.

**Table 2.** Features of AP waveforms at NG, LG and LG combined with low *K*o (LG + low *K*o) with added either sympathetic or parasympathetic activity. Baseline values of input parameters (*g*_Kr_, IVshift and *K*o) and AP waveform features are highlighted in italics.

	NG	+sympathetic act.			+parasympathetic act.		
		NG	LG	LG + low *K*o	NG	LG	LG + low *K*o
*g_Kr_*	*1*	*1*	0.7	0.7	*1*	0.7	0.7
*IVshift* (*mV*)	*0*	*0*	−3	−3	*0*	−3	−3
*Ko* (*mmol l*^−1^)	*4.0*	*4.0*	*4.0*	3.0	*4.0*	*4.0*	3.0
CL (s)	*0.92*	0.69	0.64	0.78	1.27	1.36	1.73
PP (mV)	*25.9*	26.8	27.6	27.8	22.8	24.1	23.3
APA (mV)	*87.9*	*86.6*	85.7	*88.3*	84.6	84.5	86.9
MDP (mV)	*−62.0*	−59.8	−58.7	−60.5	−61.7	−60.4	−63.6
APD_90_ (s)	*0.14*	0.14	0.17	0.17	0.14	0.17	0.16
DDR	*19.0*	21.9	23.9	18.5	10.4	9.26	6.22
DDR_100_	*40.1*	58.3	67.0	59.0	34.0	40.5	25.1

In the simulations including sympathetic and parasympathetic activity, a reduction in *K*o combined with LG causes CL prolongation, predominantly caused by decreased rates of diastolic depolarization (DDR and DDR_100_) (table 2). When combined with sympathetic activity, DDR and DDR_100_ are decreased by −23% (18.5 versus 23.9) and −22% (59 versus 67) at LG and *K*o = 3 mmol l^−1^ versus LG and *K*o = 4 mmol l^−1^. The reductions are greater, −33% (6.2 versus 9.3) and −38% (25.1 versus 40.5), when combined with parasympathetic activity.

#### 3.5.2. Randomly sampled *g*_Kr_, IVshift and potassium concentration

We used the existing 2000 random sets of input parameters *g*_Kr_, IVshift and *K*o, presented in figure 6 and evaluated the model by adding either sympathetic or parasympathetic activity. The range of CL values, obtained after adding sympathetic activity is shown in figure 8*a* (grey). For reference, the grey and red dashed lines indicate baseline CL at NG without (CL = 0.92 s) and with added sympathetic activity (CL = 0.69 s, −25%), respectively. The model produces valid periodic pacemaker activity with all sets of input parameters. The relationship between CL and *K*o at NG is further highlighted in blue and at LG (*g*_Kr_ = 0.7, IVshift = −3 mV) in red. The AP waveforms at *K*o = 3, 3.5 and 4 mmol l^−1^ are presented in figure 7*a*. There is a trend of CL prolongation with reduced *K*o overall, as well as at NG and LG. For the whole range of *K*o, CL is shorter at LG compared to NG (red data are below blue) and the difference is greatest at *K*o = 4 mmol l^−1^ (CL = 0.64 s versus 0.69 s, −7%).

The relationship between CL and *K*o when combined with parasympathetic activity is shown in figure 8*b* (grey). The grey and red dashed lines indicate baseline CL at NG without (CL = 0.92 s) and with added parasympathetic activity (CL = 1.27 s, +38%), respectively. There is a systematic CL prolongation with the reduction in *K*o. Some of the CLs are greater than 4 s, especially at lower *K*o, reaching CL approximately 25 s (data not shown). We show the distribution of the full extent of CL values on a logarithmic scale in figure 8*c*. The relationship between CL and *K*o at NG is further highlighted in blue and at LG (*g*_Kr_ = 0.7, IVshift = −3 mV) in red. The AP waveforms at *K*o = 3, 3.5 and 4 mmol l^−1^ are presented in figure 7*b*. CL is prolonged with reduced *K*o, but the prolongation is greater at LG versus NG (red data above blue), with the biggest difference at *K*o = 3 mmol l^−1^ (CL = 1.73 s versus 1.50, +15%). Some combinations of input parameters with added parasympathetic activity do not produce periodic pacemaker activity (data not presented, see below).

**Figure 9.**
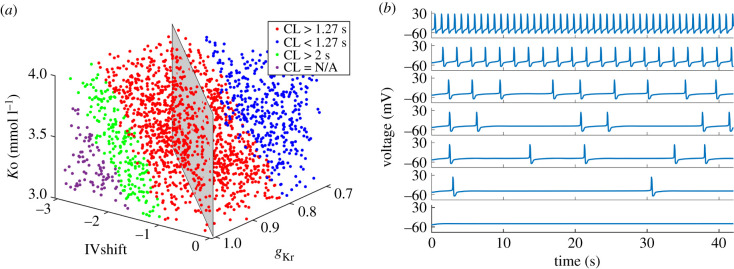
(*a*) Classification of CL values produced with randomly sampled *g*_Kr_ [0.7, 1], IVshift [−3, 0]mV and *K*o [3, 4]mmol l^−1^ when combined with parasympathetic activity. Red data indicate CL prolongation and blue data CL shortening versus baseline+parasympathetic activity (CL = 1.27 s). Green data indicate CL > 2 s and purple data invalid CL where the model did not produce periodic AP. (*b*) Examples of simulated APs: baseline (CL = 0.92 s, top row), CL = 2 s (second row), sinoatrial pauses (rows 3–6) and no periodic AP (last row). Latin hypercube sampling, *N* = 2000.

To identify input parameters which in combination with parasympathetic activity produce long CL or no pacemaker activity, we divided the parameter space as shown in figure 9*a*. Red data indicate CL prolongation and blue data CL shortening compared to NG with added parasympathetic activity (CL = 1.27 s). Green data indicate CL > 2 s and purple data indicate invalid CL with no periodic pacemaker activity. At *K*o = 4 mmol l^−1^, all simulations result in valid periodic activity, with some CLs longer than 2 s (see also figure 8*b*). These are roughly confined to the area with *g*_Kr_ > 0.9 and IVshift < −2 mV. At *K*o = 3 mmol l^−1^, this area extends to *g*_Kr_ > 0.8 and IVshift < −1 mV (green circles). At *K*o = 3.7 mmol l^−1^, the model stops producing pacemaker activity around *g*_Kr_ = 1 and IVshift = −3 mV. At *K*o = 3 mmol l^−1^, this area extends to *g*_Kr_ > 0.88 and IVshift < −1.8 mV. Figure 9*b* shows examples of APs over 40 s simulations. Baseline AP (CL = 0.92 s) is shown in the top row, followed by CL = 2 s in the second row and sinoatrial pauses in rows 3 to 5. The penultimate row shows the longest CL (around 25 s), and the last row shows an example with no periodic pacemaker activity.

## 4. Discussion

In this first study investigating the effects of hypoglycaemia on the SAN electrophysiology, we demonstrate the following findings. First, in response to hypoglycaemia, the reduced conductance of the hERG channel and the shift in its I–V relationship produce opposite effects on the pacemaker rate. When combined, they cancel each other out, resulting in a small decrease in heart rate. Second, mild hypokalaemia causes a slight decrease in heart rate. When combined with hypoglycaemia, there is a marked further decrease in heart rate. Third, hypoglycaemia combined with sympathetic activity enhances the effect of sympathetic activity by further increasing the heart rate compared to NG. However, when hypoglycaemia is combined with hypokalaemia and sympathetic activity, the effect of sympathetic activity is diminished, causing a smaller increase in heart rate compared to that at NG with added sympathetic activity. Fourth, hypoglycaemia enhances the effect of parasympathetic activity by causing a greater decrease in heart rate compared to NG. Hypoglycaemia combined with hypokalaemia and parasympathetic activity causes a further marked reduction in heart rate, resulting in sinoatrial pauses, sinus arrest and block of pacemaker activity.

Combined, the above findings indicate that hypoglycaemia tends to decrease the pacemaker rate of the SAN. During experimental hypoglycaemia, where glucose was reduced in a controlled environment using insulin infusion, hypoglycaemia counterregulation caused a significant increase in circulating adrenaline and noradrenaline concentrations together with a significant decrease in potassium in healthy people [15] and in people with type 2 [15] and type 1 diabetes [14]. Despite the significant increase in adrenaline and noradrenaline, there was a relatively small (about 3 bpm) increase in heart rate during hypoglycaemia compared to NG [14,15]. It is therefore possible that decreased glucose and potassium levels counteract the effects of the autonomic nervous system at the level of the SAN. Robinson *et al.* have reported the results of experimental studies in healthy subjects where they restored normal concentrations of potassium during both NG and hypoglycaemia [14]. They have shown that heart rate did not change during NG after potassium has been added (*K*o = 3.6 versus 4 mmol l^−1^). On the other hand, there was an approximate 7 bpm increase in heart rate (61 versus 68 bpm, +11%) when potassium was added (*K*o = 3.2 versus 3.9 mmol l^−1^) during hypoglycaemia. These data are in line with our findings that decreased potassium does not significantly affect the heart rate during NG but causes a decrease in heart rate when combined with decreased glucose. For comparable values of *K*o, our model shows a 2% higher heart rate during NG and a 9% higher heart rate during hypoglycaemia.

In our study, we demonstrate a potential mechanistic explanation for hypoglycaemia-induced bradycardia, which has been observed during clinical episodes in people with type 1 [30] and type 2 diabetes [22]. We have undertaken several observational studies, where we compared rates of cardiac arrhythmia during spontaneous hypoglycaemia versus NG. We observed increased incidences of bradycardia in people with type 1 diabetes [10], with type 2 diabetes [8] and with type 2 diabetes after discharge from ICU [11]. However, increased rates of bradycardia were only detected in a few individuals. We speculated that these effects are idiosyncratic and that mechanisms other than those involved in hypoglycaemia counterregulation might contribute to the development of bradycardia [10,11,31]. These could include autonomic dysfunction and/or abnormal and variable autonomic responses such as those observed during experimental hypoglycaemia, where the early parasympathetic withdrawal was followed by parasympathetic reactivation during sustained hypoglycaemia [15]. Nocturnal hypoglycaemia could also increase the risk as sleep and prone position diminish the sympathoadrenal responses during the night [32]. Our findings that decreased blood glucose and potassium diminish the effect of the sympathetic activity and enhance the parasympathetic activity might further exacerbate the risk of bradycardia in the above cases.

To validate the responses of our model during hypoglycaemia, we used the heart rate differences between matched spontaneous hypoglycaemia and NG episodes from our observational study in people with type 1 diabetes [10]. We chose only nocturnal episodes due to diminished sympathoadrenal responses which in turn also cause a smaller reduction in *K*o. Our computational model shows an approximate −1% decrease in heart rate during hypoglycaemia at *K*o = 4 mmol l^−1^. In our observational study, there was a nonsignificant +2% increase in heart rate during hypoglycaemia versus NG with a mean difference of 1.5 bpm (95%CI −1.3, 4.4 bpm). In a similar study, Koivikko *et al.* [33] reported a +2% increase. Our computational data are within the confidence intervals of those from the above observational studies and the difference in trends could be partly due to counterregulatory sympathetic activation.

There are several conditions which could combine with the effects of hypoglycaemia to further promote decreased pacemaker rate at the SAN. Channelopathies, such as mutations in the HCN4, SCN5A and KCNQ1 genes, which encode ion currents *I*_f_, *I*_Na_ and *I*_Ks_, can lead to reduced pacemaker rate. In addition, remodelling of the *I*_f_ depolarization current in trained athletes, which causes decreased resting heart rate and potential training-induced bradycardia [34] could lead to increased risk during hypoglycaemia. These cases could be further explored in the computational model of the human SAN as well as in observational or experimental hypoglycaemia studies.

**Figure 10.**
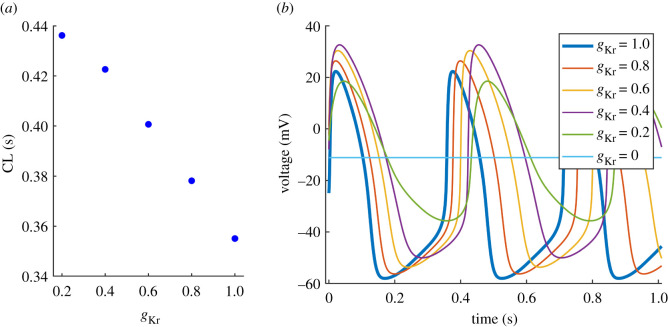
Effect of reduced *g*_Kr_ on CL in a computational model of rabbit SAN. (*a*) CL is prolonged with the reduction in *g*_Kr_. (*b*) The corresponding AP waveforms indicate APD prolongation, elevated MDP and reduced DDR. The model does not generate AP with *g*_Kr_ = 0.

In our model, we show a gradual increase in pacemaker rate with decreased *g*_Kr_ (figure 2). This contradicts experimental data from animals, where selective blockage of *I*_Kr_ resulted in decreased rates in rabbit [35] and guinea pig [36] SAN. Due to the lack of human experimental data to confirm our findings, we explored the reduction of *g*_Kr_ in a computational model of the rabbit SAN. We chose the Severi model [27], which is the parent model of the one used in this study. The AP waveforms and the relationship between *g*_Kr_ and CL are presented in figure 10. CL is proportionally prolonged with reduced *g*_Kr_ (figure 10*a*) and the pacemaker activity is abolished with complete block of *I*_Kr_ (*g*_Kr_ = 0, figure 10*b*). CL prolongation is accompanied by APD prolongation, the elevation of MDP and decreased DDR. This is in line with experimental data from the rabbit SAN [35].

In our human model, a reduction in *g*_Kr_ is accompanied by APD prolongation and elevation of MDP, as in the rabbit model. In contrast with the rabbit model, DDR increases in the human model (table 1). One of the reasons for differences between the models is the roughly 2.5 times higher resting pacemaker rate in rabbits (CL = 0.92 s in human versus 0.36 s in rabbit). While APD prolongation is similar in both models, this makes up a much bigger proportion of CL in the rabbit AP. In figure 10*b*, APD prolongations are similar to the prolongations of CL, while the opposite effects of elevated MDP and decreased DDR on CL seem to cancel each other out. By contrast, the duration of diastolic depolarization is much longer in the human model, making the CL more susceptible to changes in DDR.

To further investigate the differences between DDR responses in both models, we compared the major currents responsible for diastolic depolarization at *g*_Kr_ = 1 versus *g*_Kr_ = 0.5. In the rabbit model, the amplitude of *I*_f_ current is relatively high versus the other currents and its amplitude decreases by about 50% with reduced *g*_Kr_ (electronic supplementary material, figure S2). Similarly, the amplitude of *I*_NaCa_ markedly decreases. By contrast, the amplitude of the *I*_f_ current is much smaller in absolute terms as well as relative to the other currents in the human model (electronic supplementary material, figure S3). In addition, the amplitude of *I*_NaCa_ slightly increases with reduced *g*_Kr_. These differential responses in currents, combined with the higher resting rate in rabbits could explain the differences between the *g*_Kr_/CL relationship in humans versus rabbits. It is also possible that the decreased pacemaker rate in response to reduced *g*_Kr_ in rabbits contributes to the reduction of heart rate and occurrence of bradycardia during experimental hypoglycaemia in rats [24].

The key strength of our study is that we explored for the first time the electrophysiological effects of hypoglycaemia on the SAN. Furthermore, to mimic the physiological conditions in humans, we added the stimuli of hypokalaemia as well as sympathetic and parasympathetic stimulation. This approach overcomes some of the technical, biological and ethical constraints of *in vivo* human and animal studies. One of the limitations of the study is the lack of detailed experimental data on the effect of *Gluo* on cardiac cell electrophysiology. Zhang *et al.* demonstrated the main effects of LG on the hERG channel [17], but experimental data at finer glucose increments would greatly improve the understanding of the balance between increased heart rate, caused by reduced conductance of the channel versus slowing of the rate caused by the shift in its I–V properties and how these relate to clinically relevant glucose levels. To the best of our knowledge, there is no evidence of any other cardiac ion channel being affected by hypoglycaemia.

Fabbri *et al.* optimized their model to reproduce baseline electrophysiological characteristics from a limited number of human experimental data and validated it against baseline heart rates observed in common ion channel mutations. Given the lack of experimental data, the model is not optimized and validated for variations in heart rate and altered extracellular ion concentrations. Loewe *et al.* [37] extended the Fabbri model to work at variable *K*o concentrations by including a new formulation of *I*_Kr_, where *g*_Kr_ is dependent on *K*o. Their model showed a positive relationship between heart rate and *K*o and they found that this was in disagreement with data from the general population, which showed a negative linear relationship. Our model, as the extended Fabbri model, shows a positive relationship between heart rate and *K*o. This could mean that our model overestimates the CL at *K*o = 3 mmol l^−1^ during NG as well as with LG and in combination with parasympathetic activity. This behaviour of our model could be inherited from the parent model. Indeed, existing human atrial models show divergent and sometimes unphysiological responses to changing extracellular *K*o [38] and the corresponding electrophysiological conditions remain to be explored. Finally, the Fabbri model offers selective activation of either sympathetic or parasympathetic activity. In reality, both branches of the autonomic system are continuously active, and they complement each other to modulate the pacemaker rate. The responses of their combined activities and how these relate to their individual effects, observed in this study, remain to be investigated.

## 5. Conclusion

Computational modelling has the potential to clarify electro-pathophysiological effects of hypoglycaemia. We have shown that sinus arrest, a case of extreme bradycardia, is a plausible mechanism for SCD, possibly more probable than tachycardia during the night. Investigation of confounding factors that lead to the development of abnormal cardiac responses, as well as modelling of different cardiac cells at different levels may help to identify individuals with diabetes who are at an increased risk of sudden death due to sinus arrest during hypoglycaemia. This has important clinical implications because equivalent studies are not feasible or are challenging to perform in a clinical setting.

## Data accessibility

Numerical data, source code of the model and the code to reproduce the figures are available in a Github repository: https://github.com/AlanBernjak/Bernjak_etal_Human_SAN_model_hypoglycaemia.
